# The Tenckhoff catheter in elderly patients with chronic renal failure: placement in spinal anesthesia with open technique, without required location in the hollow of Douglas

**DOI:** 10.1186/1471-2318-11-S1-A61

**Published:** 2011-08-24

**Authors:** T Strazzullo, G Prestieri, D Di Simone, G Merola

**Affiliations:** 1 Departement of Surgical, Anaestesiologic and Emergency Sciences, University Federico II , Naples, Italy

## Background

Currently there are various techniques for catheter placement for peritoneal dialysis. The purpose of this paper is to describe our technique and our results in elderly patients.

## Materials and methods

We report our experience on the placement of the Tenckhoff catheter (TC) in elderly patients by open surgery, under spinal anesthesia, without necessarily placing the catheter in Douglas.

## Results

Between January 2004 and September 2010 we placed 42 TC in 42 patients including 25 males and 17 females between 65 and 92 years old (mean age 73 years). 7 of these patients were simultaneously subjected to another surgery to correct a defect in the abdominal wall.

Among the 29 patients still living 4 TC are no longer used: 2 for recurrential peritonitis, 1 for considerable volume polycistic kidney disease and 1 was removed due to an undefined failure.

The TC that are currently working are: 4/7 after 6 years, 1/2 after 5 years, 1/1 after 4 years, 1/1 after 3 years, 4/4 after 2 years, 9/9 to 1 years; 5/5 in the last 6 months.

In the post-operative abdomen X-rays were performed in 7 of the 26 patients who showed different locations of the distal end of the catheter: 3 in Douglas, 1 in the left side, 2 in the right side, 1 in the right upper quadrant.

## Conclusions

Placement of the TC under spinal anesthesia with open technique, not necessarily placing the tip in the Douglas, is a simple technique that guarantees good results with low operational risks and peri-operative complications, especially for elderly patients. The results of this technique in combination with concomitant hernias are particularly relevant.

## References

[B1] Chow KM Tenckhoff catheter insertion by nephrologists: open dissection technique Perit Dial Int 2010 30 5 524 7 10.3747/pdi.2009.00145 20378842

[B2] Yang PJ Mini-laparotomy implantation of peritoneal dialysis catheters: outcome and rescue Perit Dial Int 2010 30 5 513 8 10.3747/pdi.2009.00033 20190027

[B3] Mettang T Endoscopic transluminal insertion of a peritoneal dialysis catheter Perit Dial Int 2010 30 1 63 5 10.3747/pdi.2008.00193 20056981

[B4] Pantea S The placement of Tenckhoff peritoneal dialysis catheter by laparoscopic approach--our experience Chirurgia 2008 103 6 669 72 19274912

[B5] Ashegh H One-port laparoscopic technique for placement of Tenckhoff peritoneal dialysis catheters: report of seventy-nine procedures Perit Dial Int 2008 28 6 622 5 18981392

